# *Plasmodium vivax* Malaria

**DOI:** 10.3201/eid1101.040519

**Published:** 2005-01

**Authors:** Dhanpat K. Kochar, Vishal Saxena, Narvachan Singh, Sanjay K. Kochar, S. Vijay Kumar, Ashis Das

**Affiliations:** * Sardar Patel Medical College, Bikaner, Rajasthan, India; † Birla Institute of Technology and Science, Pilani, Rajasthan, India

**Keywords:** Plasmodium vivax, cerebral malaria, PCR, ARDS, renal failure, jaundice, circulatory collapse, severe anemia, hemoglobinurea, dispatch

## Abstract

We report 11 cases of severe *Plasmodium vivax* malaria in Bikaner (western India). Patients exhibited cerebral malaria, renal failure, circulatory collapse, severe anemia, hemoglobinurea, abnormal bleeding, acute respiratory distress syndrome, and jaundice. Peripheral blood microscopy, parasite antigen–based assays, and parasite 18s rRNA gene–based polymerase chain reaction showed the presence of *P. vivax* and absence of *P. falciparum*.

*Plasmodium vivax* malaria is prevalent in many regions of the world. It accounts for more than half of all malaria cases in Asia and Latin America. Despite the high prevalence of disease caused by this parasite, research into its effects has lagged disproportionately (*1*).

Organ dysfunction seen in *P. falciparum* malaria is not seen in *P.* *vivax* infections. Thus, severe malaria is reported with *P. falciparum* but not with *P. vivax* infection. If a patient with *P. vivax* exhibits severe malaria, the infection is presumed to be mixed. When patients have a mixed infection, *P. vivax* may lessen the effect of *P. falciparum* and cause the disease to be less severe. Luxemburger et al. observed that severe malaria is 4.2 times less common in patients with mixed *P. falciparum* and *P.* *vivax* infections than in those with *P.* *falciparum* alone (*2*).

## The Study

During the post-rainy season epidemic of malaria from August to December 2003, many persons along the Indonesia–Pakistan border had severe malaria caused by *P. vivax*. During the last few outbreaks, we made similar observations, but in 2003 the number of cases was comparatively higher. Clinically severe cases and complications of malaria are commonly due to *P. falciparum* and not to *P. vivax*. Beg et al. reported a patient from Pakistan with central nervous system (CNS) involvement with *P. vivax*, in which the diagnosis was confirmed by polymerase chain reaction (PCR) studies. Beg et al. reviewed the *P. vivax* cases with CNS involvement reported before 2002; however, most were diagnosed by examination of peripheral blood films (PBF) (*3*).

We searched available literature and could find only isolated reports of severe *P. vivax* malaria with cerebral malaria, thrombocytopenia, disseminated intravascular coagulation (DIC), acute respiratory distress syndrome (ARDS), and renal involvement caused by *P. vivax*. In most cases, the diagnosis was made by PBF examination without molecular diagnostic confirmation, thus allowing for potential errors in species diagnosis (*4*–*13*). Although detection of *P. vivax* in PBF is the standard, its presence does not rule out undetected mixed infection. To rule out this possibility, all the patients received a thorough diagnostic evaluation, which included PBF examination, a rapid diagnostic test for malaria (OptiMAL test, DiaMed AG, Switzerland, which is based on detecting specific *Plasmodium* LDH antigen by using monoclonal antibody directed against isoforms of the enzyme), and PCR. Our findings are shown in Tables 1 and 2.

**Table 1:** Clinical characteristics of severe vivax malaria patients

Patient No.	Age (y)/sex	Clinical presentation*	Parasitemia (density) (*P. vivax*/mm^3^)	Diagnostic tests for malaria			Other relevant information	Outcome
				PBF	RMDT OptiMAL test†	PCR		
1	30, F	ARDS	6,000	+	Positive	+	Skiagram chest suggestive of pulmonary edema	Died
2	17, M	Renal failure, bleeding diathesis	20,000	+	Positive	+	PT >60 (Control – 16) BT, CT, PT – N	Recovered
3	53, M	Jaundice‡	35,000	+	Positive	+	Epistaxis, hemoglobinurea BT, CT, PT – N	Recovered
4	20, F	Cerebral (GCS– 3) anemia, ARDS, PCF	15,000	+	Positive	+	BP <70 mmHg (systolic) CSF – N CT scan head – could not be done	Died within 5 of admission
5	45, M	Renal failure, jaundice‡	36,000	+	Positive	+		Recovered
6	22, F	Cerebral (GCS – 6) anemia	8,000	+	Positive	+	Puerpural period 3^rd^ gravida CSF – N CT scan head – N	Recovered Baby died on 14th day at residence
7	18, F	Cerebral (GCS – 5) anemia	10,000	+	Positive	+	Primigravida CSF – Normal CT scan head – N	Recovered PMNS - Psychosis Premature delivery Baby survived
8	28, F	Renal failure, ARDS, PCF	44,000	+	Positive	+	Gross hematuria BP <70 mm Hg systolic	Recovered
9	25, F	Jaundice‡, haemoglobinurea	90,400	+	Positive	+	Secondgravida	Recovered Pregnancy continued
10	50, M	Jaundice‡	18,000	+	Positive	+		Recovered
11	18, F	Renal failure, anemia, pulmonary edema	34,000	+	Positive	+	Skiagram chest – pulmonary edema	Recovered Underwent hemodialysis

*All patients were fully conscious except patients 4, 6, and 7. ALT, alanine aminotransferase; ARDS, acute respiratory distress syndrome; AST, aspartate aminotransferase; BT, bleeding time; CT, clotting time; CT, computerized tomography scan of head (non-contrast); F, female; GCS, Glasgow coma scale; LDH, lactate dehydrogenase; M, male; N, normal; PBF, peripheral blood film; PCR, polymerase chain reaction; PMNS, post malarial neurologic syndrome; PT, prothrombin time; TLC, total leukocyte count; RMDT, rapid malaria diagnostic test. †Positive for *Plasmodium vivax*, *malariae*, or *ovale* because of common LDH isoenzyme antigen and negative for *P. falciparum*. ‡Relevant investigations were done to rule out viral hepatitis and leptospirosis in all patients with jaundice.

**Table 2:** Hematologic and biochemical characteristics of severe vivax malaria patients*

Patient Number	Hb (g%)	TLC (mm^3^)	Platelet count (lac/mm^3^)	Blood sugar (mg%)	Blood urea (mg%)	Serum creatinine (mg%)	Serum bilirubin total/conjugated (mg%)	Serum ALT (IU/L)	Serum AST (IU/L)
1	7	10,000	92,000	94	24	1.0	1.0/0.4	40	30
2	8	5,800	100,000	90	95	3.5	0.9/0.3	30	27
3	9.5	5,800	50,000	105	54	1.0	10.3/6.4	360	278
4	5	6,600	120,000	60	70	1.7	2.6/1.2	36	32
5	10	9,000	87,000	120	90	3.0	3.1/2.2	39	33
6	6	8,400	80,000	80	25	0.8	0.9/0.3	25	36
7	6	9,000	96,000	60	24	1.0	1.0/0.4	40	30
8	8.4	6,000	150,000	76	60	3.0	1.6/0.6	90	80
9	9.0	8,400	75,000	138	72	0.6	16/10.9	510	546
10	7.2	8,100	110,000	80	48	0.8	4.0/3.0	187	136
11	6	10,000	121,000	90	82	4.5	1.0/0.3	36	32

*Hb, hemoglobin; TLC, total leukocyte count; ALT, alanine aminotransferase; AST, aspartate aminotransferase.

All patients were admitted to an intensive care ward dedicated to malaria control. Clinical, biochemical, and radiologic examinations were conducted to establish the diagnosis. Severe malaria was categorized and a treatment regimen of intravenous quinine was instituted according to World Health Organization guidelines (*14*). Formal approval of the hospital’s ethical committee and consent of the patients were obtained for further studies.

The PCR studies were targeted against the 18S rRNA gene of the parasite and were based on conditions reported earlier (*15*) utilizing 1 genus-specific 5′ primer and 2 species-specific 3′ primers in the same reaction cocktail. Some of the primer sequences were modified for this study: 1) 5′ATCAGCTTTTGATGTTAGGGT ATT 3′–genus specific, 2) 5′ TAACAAGGACTTCCAAGC–*P. vivax* specific, and 3) 5′GCTCAAAGATACAAATATAAGC 3′–*P. falciparum* specific (Figure). Our PCR results in each sample ruled out the possibility of coinfection with *P. falciparum*. Each sample was subjected to a minimum of 4 rounds of PCR with varying template amounts to eliminate the possibility of overlooking *P. falciparum* coinfection. In this report, we have not included 2 samples that showed *P. vivax* infection in PBF examination but showed evidence of mixed infection in PCR examination. The result of PCR analysis of 1 sample is shown in lane 8 of the Figure.

**Figure:**
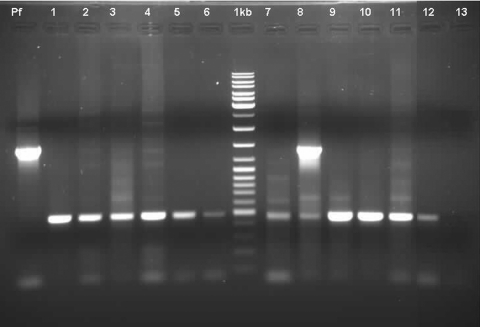
Polymerase chain reaction analysis of patient samples: lane Pf = positive control showing *P. falciparum* band at position ≈1400 bp; lanes 1–7 and 9–12 = *P. vivax*– positive samples showing band at ≈500 bp; (lanes 1–7 correspond to cases 1–7, and lanes 9–12 correspond to patients 8–11 numbered in Tables 1 and 2.); lane 8 = sample showing bands at ≈1,400 bp and 500 bp, indicating mixed infection; lane 13 = negative control, normal human DNA; lane M = 1-kb DNA ladder mix (MBI Fermentas, SM#033)

## Conclusions

The essential pathologic feature of severe malaria is sequestration of erythrocytes that contain mature forms of the parasite in the deep vascular beds of vital organs, thus producing cerebral malaria, renal failure, hepatic dysfunction, or ARDS. However, severe anemia and thrombocytopenia that causes bleeding diathesis is produced by hemolysis, reduced cell deformity of parasitized and nonparasitized erythrocytes, increased splenic clearance, reduction of platelet survival, decreased platelet production, and increased splenic uptake of platelets, and can be produced by *P. vivax* and *P. falciparum* infection. Our clinical data from these patients strongly indicate that *P. vivax* can cause both sequestration-related and nonsequestration-related complications of severe malaria, including cerebral malaria, renal failure, circulatory collapse, severe anemia, hemoglobinurea, abnormal bleeding, ARDS, and jaundice, all of which are commonly associated with *P. falciparum* infections. None of the patients described in this study had evidence of *P. falciparum* infection at the level of antigen (parasite LDH) and 18S rRNA–based PCR test, apart from PBF examination.

This is the first detailed report of severe *P. vivax* malaria. We cannot comment on a pathogenic mechanism causing multiple organ dysfunction and the characteristics of host-parasite interrelationship responsible for it. A detailed prospective study is required to address these issues.
